# Helps in Study

**Published:** 1883-07

**Authors:** George Watt

**Affiliations:** Xenia, O.


### Helps in Study.

BY GEORGE WATT, XENIA, O.

[Read before the Mad River Valley Dental Society.]

A rural blacksmith's little boy watched his mother as she was tacking down her carpet, at the close of that joyous season, known as house-cleaning week. He saw how nicely each little nail went to its place, under the blows of the small hammer in the hand of the skillful woman. He was a kind boy, and wished to help in the work; but considering his own smallness, the thought struck him that he would need a much larger hammer than the one used by his mother, in order that his blows might equal hers. He did'nt succeed very well, even though he tried his father's sledge-hammer; and sometimes professional men have but little better success when they resort to similar devices.

Take an illustration. Is there more than one variety of dental caries is a question that has been asked (and answered, too, for that matter, but an effort has been made in a certain quarter, to re-open it). Now do we need a microscope, balances, re-agents, etc., to settle this question? Certainly not; for the unassisted eye, and ordinary observation, will answer all purposes. We find one specimen of decay jet black, even when recent. It progresses slowly. It changes the texture of much tooth-tissue that is not displaced or removed. It leaves this changed tissue brittle and friable, with but little sensitiveness in the bottom of the cavity. It is mainly found in the teeth of persons whose breath ordinarily gives off sulphuretted hydrogen. And all these can be recognized without microscope and scales as well as with them. There is no call for them unless to make a display.

Another specimen of decay is found, which, new or old, is whiter than the texture of the sound tooth. This usually is deeper in proportion to its diameter than the black; the bottom of the cavity is much more sensitive; more of the tooth-substance is removed or displaced; the decayed portion is plastic rather than friable; very much of the decay can be washed or wiped out of the cavity; it makes rapid progress. And all these points of difference can be recognized as readily without as with instruments or methods of special research.

Or suppose the question to be, are the lime-salts—the hard materials of the tooth—removed by dental caries? The believer in the chemical theory of decay will tell us that the question is indefinite, but that, if this form of it must be retained, he will answer both yes and no. He will tell us that when he finds a jet black decay, in its early stages, he does not expect to find much, if any diminution of the quantity of lime-salts. What is there to remove them? He does not believe that the re-agent concerned is capable of forming soluble compounds with the lime-salts of the tooth, or at most, they can be but slightly soluble. And besides, they are retained in a state of minute division among the blackened organic matter, which greatly tends to keep them in place. Even were they readily soluble in saliva or mucus, the carbonized organic matter would almost exclude the solvent and thus allow them to remain. The intelligent operator would readily recognize their presence by physical signs, while he would also realize the fact that there had been nothing in contact capable of removing them.

Or he will tell us he finds a cavity of decay greatly differing from the black one in several respects. It may be almost colorless, a slightly yellow, orange, brown, or even black, according to its age, and perhaps influenced by some other circumstances. It is far more common than the jet black variety, which, however, is by no means uncommon. But its leading trait is that the cavity of decay is always partly, and often almost completely, filled with soft tissue, which is not readily washed or wiped out, as is the debris in white decay, but is easily cut, even when so little disintegrated that its organic structure is still quite observable.

Now, in this variety, the operator needs no chemical analysis to inform him that very much, indeed nearly all, of the hard matter is removed. And a chemist cannot persuade him to the contrary by the most careful analysis of a specimen of decay, especially if he fails to tell which kind of caries he had under examination, or if he incidentally mentions that it was the black variety. And when excavating a cavity of this gelatinous variety of decay (if the term can be tolerated), the intelligent dentist knows as well, without any analysis, as the best chemist can know with it, that the hard matter of the tooth is mainly dissolved away, as far as the decay has progressed. The chemist can tell more accurately whether or not a trace of lime is left; but the facts necessary to proper practice can be recognized by the one as well as by the other.

It cannot be denied that, while chemical and microscopic researches are, in their very nature and essence, extremely valuable, there is a degree of pedantry sometimes displayed in their use, by parties not competent to use them properly or to make proper deductions from the results thereby obtained. We can illustrate this by narrating an incident which occurred in an early period of our medical practice: A man as strong as a wild buffalo fancied he had contracted pulmonary consumption. He had heard of the stethoscope, and demanded an examination by its use. His physician had none, and borrowed ours, politely asking us to accompany him and witness the examination. The instrument was not so common then as it is now. Our curiosity had become aroused, and we went with him. After dilating on the merits of the instrument, and especially on the accuracy of its revelations, he began the examination. Placing one end of the instrument to the patient's mouth, and the other to his own nose, he directed the patient to breathe through the mouth only, and he smelled the breath as it emerged from the instrument. He pronounced the lungs perfectly sound, and the patient was happy.

This is an extreme case, but it is approximated by some of the so-called researches within our own profession.

Now, it must not be supposed that we lightly estimate chemical analysis and microscopic research when they are legitimate, that is, when they are used for the purpose of gaining useful knowledge, and not for display. A man had a severe attack of erysipelas, which had begun on the tip of his nose. He believed that all the trouble had resulted from the bite of a spider, and he became the relentless enemy of spiders. One morning he saw one draw itself up to the ceiling, and at once he seized his trusty rifle and shot it, without mercy. In our boyish way of estimating, we thought the gun quite uncalled for, as a broom or a stick would have served as well; but either would have been less sensational, and much less noisy than the warlike weapon. We are often reminded of this when we see instruments brought to bear in research which can be made quite as well, if not better, without them. When the microscope and the balances are brought into requisition to know if any considerable proportion of the lime-salts is removed from a carious cavity, by that form of decay which is characterized by the presence of large quantities of gelatinous materials in the cavity, we are shooting spiders with a rifle, if not smelling breaths through the stethoscope.

An amusing feature, too, in some of these so-called instrumental researches, is found in the fact that, in a few years, or even after a few months, in some cases, the observers assume that, of all "our glorious prefession, they, and they only, are experimenters, that only they are making progress." They tell aged men their experience in the most patronizing way; and, in some cases find, afterward, that their reports of their experiments read so much like the aged brethren's accounts of similar ones, tried before they were born into the profession, that they are in danger of being charged with plagiarism. Sometimes a compound word is coined for the occasion by the youthful experimenter to express his own opinion that the more aged member can merely talk about the researches of others, but has made none himself, while the truth is, in some cases, that he has tried more experiments than the young brother has ever heard of. But all this is in perfect accord with human nature, and we can see the principle illustrated almost daily. Not long ago we heard a student, who had spent less than six months in a dentist's laboratory, talking with a mechanical dentist of long experience, advising him to come and learn from him, saying: "If you only understood dentistry as well as I, you could double your wages at once." But even this consideration failed to induce the old man to take a course of instruction from the would-be young teacher. And this is human nature, too, for we old men often neglect, or even reject opportunities for improvement.

The microscope and chemical analysis have each an important office to fill in dental science, and it is not likely either will be too highly esteemed. All we ask is that they be legitimately used by competent parties. If dental caries results, to any extent, from the action of acids, it is very important to know what acids. And here the microscope comes in as an important help. If an acid combines with tooth substance, a salt, or salts must result; and the quantity obtainable for examination in any given case must be small. Much can be learned by watching, with suitable power, the crystallization and re-crystallization of the salts thus formed. By evaporating the liquid slowly, and again more rapidly, by using different powers, and sometimes by polarization of the results, much information may be gained. We have found such observations so fascinating that very often we have chased night into morning, day after day for many weeks in succession, while making them.

The experimenter often fails to get the best possible aid from the microscope by using too high powers. In the popular mind this instrument is valuable, or otherwise, in proportion to its magnifying powers, while the distinctness of its definings are left out of consideration, or regarded as of minor importance. How often, when a microscope is under consideration, the first question is, how much does it magnify? And even some, who regard themselves as experts, failed to give the most reliable information, from having relied almost exclusively on high powers. When we bear in mind that, other things being equal, the liability to mistake in observation varies in the ratio of the squares of the powers used, we can see the importance of receiving such information with a few grains of allowance. High power observations are very important, as well as instructive. But what we are asking for is reasonable caution in the statements of the results, repeated observations, also, before positive assertions are made respecting them, and due caution, as well, in the reception of the information thus obtained. To steer straight forward, avoiding blind credulity, on the one hand, and cold scepticism on the other, should be the aim of every progressive mind in our profession. Truth, simple, pure, and bright, is found only in this way. May you all attain to it, and enjoy the bliss which can not be otherwise possessed.

